# Transcriptome analysis of *Taenia solium *cysticerci using Open Reading Frame ESTs (ORESTES)

**DOI:** 10.1186/1756-3305-2-35

**Published:** 2009-07-31

**Authors:** Carolina R Almeida, Patricia H Stoco, Glauber Wagner, Thaís CM Sincero, Gianinna Rotava, Ethel Bayer-Santos, Juliana B Rodrigues, Maísa M Sperandio, Antônio AM Maia, Elida PB Ojopi, Arnaldo Zaha, Henrique B Ferreira, Kevin M Tyler, Alberto MR Dávila, Edmundo C Grisard, Emmanuel Dias-Neto

**Affiliations:** 1 Laboratório de Neurociências (LIM27), Departamento e Instituto de Psiquiatria, Faculdade de Medicina da Universidade de São Paulo, Rua Dr. Ovídio Pires de Campos 785, CEP 05403-010, São Paulo, SP, Brazil; 2 Laboratórios de Protozoologia e de Bioinformática, Departamento de Microbiologia, Imunologia e Parasitologia, Universidade Federal de Santa Catarina (UFSC), Caixa postal 476, CEP 88040-970, Florianópolis, SC, Brazil; 3 Laboratório de Biologia Molecular e Bioinformática, Área das Ciências Biológicas e da Saúde, Universidade do Oeste de Santa Catarina (UNOESC), CEP 89600-000, Joaçaba, SC, Brazil; 4 Departamento de Ciências Básicas, Faculdade de Zootecnia e Engenharia de Alimentos. Universidade de São Paulo (USP), Av. Duque de Caxias Norte, 225, Centro, CEP 13630-000, Pirassununga, SP, Brazil; 5 Departamento de Biologia Molecular e Biotecnologia and Centro de Biotecnologia, Universidade Federal do Rio Grande do Sul (UFRGS), Av. Bento Gonçalves 9500, Prédio 4342131, Setor IV, Campus do Vale, CEP 91501-970, Porto Alegre, RS, Brazil; 6 Biomedical Research Centre, School of Medicine, Health Policy and Practice, University of East Anglia (UEA), NR4 7TJ, Norwich, Norfolk, UK; 7 Instituto Oswaldo Cruz, Fundação Oswaldo Cruz (FIOCRUZ), Pavilhão Leônidas Deane, Avenida Brasil 4365, CEP 21040-360, Rio de Janeiro, RJ, Brazil; 8 Department of Genitourinary Medical Oncology, MD Anderson Cancer Center, 1515 Holcombe Blvd., Unit 1374 Medical Center, 77030 Houston, USA

## Abstract

**Background:**

Human infection by the pork tapeworm *Taenia solium *affects more than 50 million people worldwide, particularly in underdeveloped and developing countries. Cysticercosis which arises from larval encystation can be life threatening and difficult to treat. Here, we investigate for the first time the transcriptome of the clinically relevant cysticerci larval form.

**Results:**

Using Expressed Sequence Tags (ESTs) produced by the ORESTES method, a total of 1,520 high quality ESTs were generated from 20 ORESTES cDNA mini-libraries and its analysis revealed fragments of genes with promising applications including 51 ESTs matching antigens previously described in other species, as well as 113 sequences representing proteins with potential extracellular localization, with obvious applications for immune-diagnosis or vaccine development.

**Conclusion:**

The set of sequences described here will contribute to deciphering the expression profile of this important parasite and will be informative for the genome assembly and annotation, as well as for studies of intra- and inter-specific sequence variability. Genes of interest for developing new diagnostic and therapeutic tools are described and discussed.

## Background

*Taenia solium*, the pork tapeworm, infects around 50 million people worldwide and is one of the foremost public health problems in developing countries [1,2]. The high influx and immigration of people coming from endemic areas to more industrialized nations has produced a complex spreading pattern for cysticercosis which is now a world-wide issue [2].

Cystercercosis arises from the development of *T. solium *cysticerci in soft tissues as a result of ingesting *T. solium *eggs [3-5]. Neurocysticercosis; which can cause epileptiform attacks, headaches, learning difficulties and convulsions; is considered the primary cause of acquired epilepsy and its clinical/therapeutic management is difficult, highlighting the importance of search for new drug targets [6-8]. In this work, we investigate for the first time the gene expression profile of *T. solium *in the larval form responsible – the cysticerci.

High throughput sequencing for gene discovery and gene expression profiling using traditional [9] or alternative 'Expressed Sequence Tags' (ESTs) such as 'Open Reading Frame ESTs' (ORESTES) [10,11] has greatly increased our knowledge of the set of expressed genes of some important helminthic parasites, notably *Schistosoma mansoni *[12,13] and its intermediate vector *Biomphalaria glabrata *[14], *S. japonicum *[15] and the cestodes *Echinococcus granulosus *[16], *E. multilocularis *[17] and *Mesocestoides corti *[18].

Recently, Aguilar-Díaz *et al*. [1] described the *T. solium *genome initiative designed to unravel the parasite's complete genome. The availability of transcribed sequences, such as those presented here, will be key to the facilitate genome annotation and gene discovery in *T. solium*.

## Results

Here we present the sequencing and analysis of 2,857 ORESTES derived from *T. solium *cysticerci, revealing a fraction of the parasite transcriptome. A total of 1,520 high-quality ORESTES generated here were deposited in dbEST database of GenBank , being 1,180 annotated as from *T. solium *[GenBank:EX150322 to EX151133 and GenBank:FD661301 to FD661668] and 340 corresponding to pig-derived sequences [GenBank:EX151134 to EX151473]. These sequences are also available at the STINGRAY system on the BiowebDB consortium website , together with relevant annotations and additional files. A list of the *T. solium *ORESTES and their respective GenBank accession numbers is presented on the Additional file 1.

### General Features

A general overview of the *T. solium *ESTs generated here is presented in Table 1. More detailed analysis of the parasite transcriptome, such as codon usage and G+C content, can be obtained online at the STINGRAY system .

**Table 1:** General features of *Taenia solium *cysticerci cDNA sequences.

Total number of valid ORESTES*	1,180
Total number of non-redundant sequences	812
Average length of non-redundant sequences	355 bp
Clusters	185
Singlets	627
Non-redundant sequences G+C average content	49%
Number of non-redundant sequences hits on/with:	
- Blast	424 (52.1%)
- RPSBlast	150 (18.4%)
- InteProScan	74 (9.0%)
- HMMER	97 (12.0%)
- *T. solium *Genome ESTs	107 (13.2%)
Sequences with no hits (blast/Interpro/HMMER)	350 (43.0%)
Number of manually validated CDS	191 (23.5%)
CDS G+C content average	53%
Number of annotated sequences	191 (23.5%)

* After trimming by quality, length and rRNA/mtRNA, as described in the text.

A total of 2,857 clones were sequenced and, after removal of poor quality (Phred<15 and/or less than 100 bp) and less informative sequences (typically rRNA and mtRNA), the remaining 1,520 ORESTES were used for sequence assembly following detailed analysis by the STINGRAY. After assembling, sequences were arranged into distinct sets named 'Cysticerci' and 'Cysticerci PIGS', which are available at STINGRAY.

The 'Cysticerci' project  corresponds to the parasite-derived transcriptome and contains a total of 1,180 ESTs clustered in 812 non-redundant sequences (185 clusters + 627 singlets), with an average size of 355 nt, totaling 288,496 nt.

The 'Cysticerci PIGS' dataset  was determined on the basis of blast similarity analysis with high scores against genomic sequences of *S. scrofa*. It is composed of 340 non-redundant singlets with an average size of 390 nt and about 132,000 nt in total. The stringency criterion used here warrants that most of this subset is certainly composed of the host transcripts, which may include transcripts relevant for the host-parasite interaction.

### The parasite transcriptome

Among the parasite's 812 non-redundant sequences (627 singlets + 185 clusters), 462 yielded significant hits with at least one of the databases used for comparative analysis . From these sequences with significant hits, 204 showed similarity with sequences of parasitic metazoan species, including sequences from the *T. solium *Genome Project (84), from other *Taenia *species such as *T. saginata *(2), *T. crassiceps *(2), *T. asiatica *(1), as well from other parasitic cestodes as *E. granulosus *(7) and *E. multilocularis *(4). Hits were also found against sequences from platyhelminths such as *S. mansoni *(88), *S. japonicum *(11), *Clonorchis sinensis *(1) and *Fasciola hepatica *(6). The remaining 263 sequences showed similarity with other metazoan species (see Additional file 2). For 350 sequences no hits were obtained on Blast, InterProScan or HMMER analyses.

After automated and manual annotation of all 812 non-redundant sequences, 191 were validated as coding sequences (CDS) (Table 1), of which 60 were considered hypothetical proteins or hypothetical conserved proteins. The number of ORESTES sequences according to their annotation identifiers is given in Additional file 3. As expected, this dataset enriched for coding sequences and showed a higher G+C content (53%) as compared to the total dataset (49%) (Table 1).

**Figure 1:**
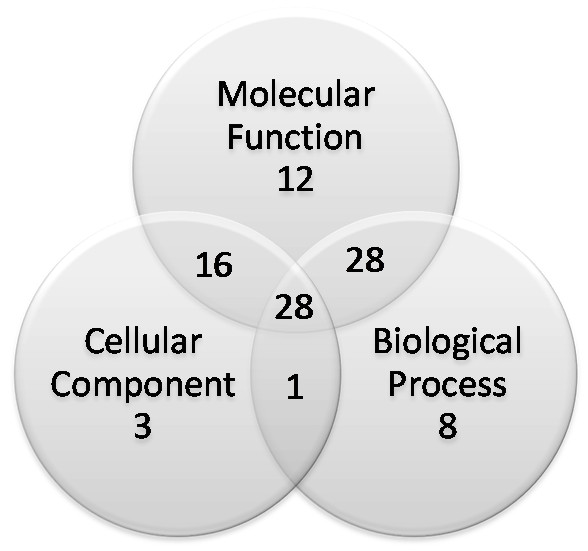
**Curated Gene Ontology categorization of the *Taenia solium *transcriptomic sequences**. Graphs shows the number of *T. solium *ORESTES sequences with curated Gene Ontology categorization of the distinct ontologies, Biological Process, Molecular Function and Cellular Component.

**Figure 2:**
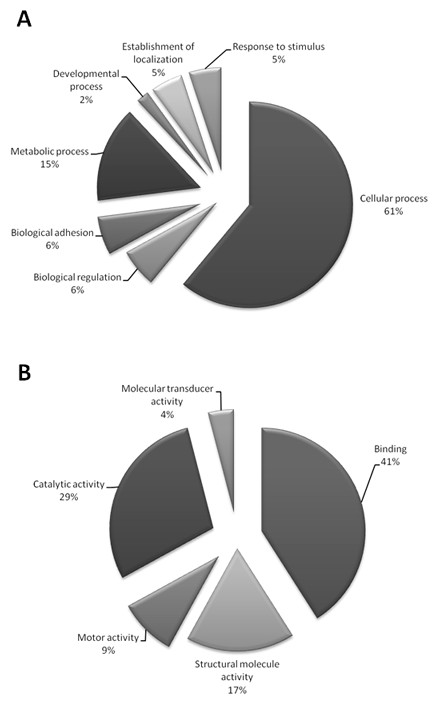
**(A) Distribution of the *Taenia solium *sequences according to the Gene Ontology sub-categorization concerning Biological Process**. The predominant category of the Biological Process ontology was Cellular Process in *T. solium *ORESTES sequences. **(B) Distribution of the *Taenia solium *sequences according to Gene Ontology sub-categorization Molecular Function**. Among the Molecular Function Ontology it was possible to attribute the Binding and Catalytic activity function terms to sets of sequences, indicative of a close relationship between host and parasite gene regulation during the process of cyst establishment and the host response to infection.

Analysis of the 191 annotated sequences using Gene Ontology (GO) allowed the categorization of 96 sequences, among which 84 were classified according to molecular function, 65 to biological processes and 48 to cellular component, several with multiple categories (Fig. 1). From the 65 sequences with biological processes annotation, the most frequent GO sub-categories were proteins related to cellular processes (40), followed by metabolic processes (10), biological regulation (4) and adhesion (4) (Fig. 2A). Among the GO molecular function sub-categories, binding (34), catalytic activity (24), structural molecule activity (14) and motor activity (7) were the most frequent (Fig. 2B). It is noteworthy that a relevant fraction of the transcripts revealed here appear to be related to structural aspects (such as adhesion, binding or structural molecule activity) that might be involved with the solid constitution of the cysts and their establishment on host tissues (see Additional file 4). A detailed description of each GO sub-category can be found on the annotated database available at the STINGRAY system .

The search for predictive sub-cellular localization of the products related to each annotated CDS was performed using the Wolf-PSORT software [19] and returned 113 hits. Among the 13 sequences with predictions of having extracellular localization with high scores (>25), only seven have a putative function assigned by GO. Among these, three are probably not extracellular ('40S Ribosomal protein S19' [GenBank:EX150987], 'Deoxyribonuclease I' [GenBank:EX151091], and 'Sec61-like protein' [GenBank:EX150487]), two may have extracellular localization ('WD40 repeat' – [GenBank:EX150998] and 'Heat-shock protein' [GenBank:EX151058]), while the 'TolA protein' [GenBank:EX150587] and the 'Cadherin family member (cdh-4)' [GenBank: EX150991] are probably extracellular (see Additional file 5). Considering the Wolf-PSORT limitations in predicting cellular localization based on short sequences such as ESTs and the fact that none of the 113 proteins predicted as extracellular were annotated as antigens, even though most *Taenia *sp. proteins already reported in the literature have precisely that description, further analysis using full length sequences are necessary to confirm these results.

### Conserved Domains and Motifs

The search for protein motifs among the parasite sequences was performed by similarity searches using InterProScan and RPSBlast using all databases available on the STINGRAY system and pointed out 64 distinct motifs distributed in 79 non-redundant sequences (see Additional files 6, 7 and 8). Among these, the 'pistil-specific extensin-like protein motif' [GenBank:IPR003882] was observed in 16 sequences, the 'vinculin/alpha-catenin' [GenBank:IPR006077] in 12, the 'glutelin' [GenBank:IPR000480] in six, 'fibronectin type III-like fold' [GenBank:IPR008957] in five and several others with four or less hits. Detailed categorization of the *T. solium *sequences according to the Eukaryotic Orthologous Groups (KOG) categories is shown on the Additional file 9.

A sensitive search for protein family recognition using multiple alignments was carried out with HMMER software and revealed 92 sequences of our parasite dataset generating at least one hit with the Pfam HMM profiles library. A domain with a still unknown function (DUF1787) was found in 12 sequences, the 'PT-PT repeat' in another nine sequences, the 'Hsp20/alpha crystallin family' (HSP20) and the 'I-set-immunoglobulin' (I-set domains) in four, the 'spectrin repeat' (SPECTRIN) and 'EGF-EGF-like domains' in another three sequences.

### Comparisons with taeniid sequences

Only 117 of the 812 *T. solium *cysticerci clustered sequences described in the present study revealed similarity with the *T. solium *Genome Project ESTs available at GenBank. Among these 117 sequences, 107 showed similarity on tblastx and 100 on blastn analysis with ESTs of the *T. solium *Genome Project, 39 with exclusive hits to the larval stage sequence, 11 with the adult stage and 67 with genes expressed in both life-cycle stages (see Additional file 10).

Except for nine sequences from *Taenia *sp. or *Echinococcus *sp., the remaining cestode-related sequences presenting high score (>90) on blast against the *T. solium *sequences described in this study, were from *Mesocestoides corti *(heat shock 70 kDa protein) and from *Diphyllobothrium dendriticum *(actin). Further 36 low-score (<90) hits with the 28S ribosomal RNA from distinct cestode species were observed.

Comparative analysis against *E. granulosus *sequences from GenBank mainly revealed constitutive genes such as actin, paramyosin and others metabolic enzymes. However, two clusters [GenBank:EX151048, GenBank:EX151014] showed high similarity with genes coding for ERM family proteins (ezrin, radixin, moesin), exclusively with EST from larval stage of *T. solium *(see Additional files 6 and 8). Some of these proteins were characterized in *Echinococcus *species and received distinct names such as EM10, EG10, EM4 and antigen II/3, despite their high nucleotide similarity. In *E. granulosus *and *E. multilocularis *these antigens are basically found in the germinal layer of brood capsules and in the tegument of protoscolices, associated with larval stage. Gonzales et al. 2007 [20], showed that the TEG-Tsag gene of *T. saginata *is homologous to EM10 and EG10 genes of *Echinococcus *spp. and 97% identical to its *T. solium *homologue. However, alignment of this *T. solium *gene with the two clusters sequences described in the present study [GenBank:EX151048, GenBank:EX151014] clearly showed high sequence variability, despite the conserved blocks. The TEG molecules are characterized by an N-terminal FERM domain and a C-terminal ERM domain which are found in a number of cytoskeletal-associated proteins located at the interface between the plasma membrane and the cytoskeleton and in proteins interacting with lipid membranes. Thus TEG protein may play a role in tegument function and interaction with the host.

### Genes of interest

A number of transcripts identified here could be of interest for further study (see Additional file 11). At least 30 genes coding for proteins potentially involved in parasite development, including transcriptional factors, component of chromatin remodelling complexes, cell adhesion-related molecules, receptors and other transducing components of signalling pathways have been identified. Moreover, putative orthologues of two proteins possibly associated to invertebrate immunity were identified for the first time in *T. solium *cysticerci: a 'heat shock 90 kDa protein' [GenBank:EX150676] and an 'anaphylatoxin-like domain protein' [GenBank:EX150322, GenBank:EX150873].

Fifty-one *T. solium *ORESTES revealed similarity with known antigens, including five previously characterized helminth antigens with potential for development of immunodiagnostic and/or vaccines. These are 'paramyosin' [GenBank:EL745866, GenBank:EL750686, GenBank:EL762552], 'major egg antigen' [GenBank:EL740635, GenBank:EL758824, GenBank:EL760346], 'cathepsin L-like cysteine proteinase' [GenBank:EL742569], 'heat shock 70 kDa protein' [GenBank:EL740975, GenBank:EL740984, GenBank:EL741400, GenBank:EL744008, GenBank:EL744338, GenBank:EL745376, GenBank:EL747548, GenBank:EL747588] and the 'H17g' or 'TEG-Tsol surface antigen' [GenBank:AJ581299], which is highly conserved among *T. solium *and *T. saginata*.

## Discussion

Transcriptome investigations have greatly benefited from the recent maturation of gene expression approaches. Among these, the microarray has evolved as the most prominent high-throughput method to assess a given expression profile. However, they are still subjected to hybridization issues such as reaction kinetics and probe mismatches as former methods. Also, microarrays cannot adequately address expression profiles of samples containing mixed species, which are yielded in studies of most parasite interactions. In these situations, the use of short gene tags, such as SAGE [21] is also problematic, due to the ambiguous tag-to-gene assignment and the difficulties of gene identification, especially in situations when the genome and/or the transcriptome of one of the species is not available. By comparison, the generation of longer sequence tags, such as those derived from EST or ORESTES, can facilitate gene discovery and annotation and also provides a much less ambiguous tag-to-gene mapping.

As formerly shown, ORESTES is able to give a normalized transcriptome view, as well as to characterize sequences from the central portion of the genes, including the less-abundant transcript markers [10,11,22-24]. The normalization capability of ORESTES, together with its ability to sample the central portion of genes makes this approach complementary to traditional ESTs, more frequently used in large-scale cDNA sequencing projects. Thus, as we have shown before for other species, including humans [11], *S. mansoni *[12,13], *Drosophila melanogaster *[25] or *Apis melifera *[26], ORESTES provides a distinct contribution to gene discover in *T. solium*. The present study shows the first comparative sequence analysis of the *T. solium *transcriptome using ORESTES from the larval stage (cysticerci).

Comparison of the *T. solium *ORESTES generated in this study with all *T. saginata *and *T. solium *sequences retrieved from GenBank showed identical hits with both datasets, indicating a high level of conservation in genes like 'Tsp36 small heat shock protein'. Few hits were obtained from other taeniids (*T. crassiceps *and *T. asiatica*), which may be due to their small sequence datasets or to the higher distance from *T. solium *and these other species. As an example of such intra-genus variability, *T. asiatica *is morphologically similar to *T. saginata *occurring in almost all Asian countries being capable of infecting pigs and humans [27] possibly leading to cysticercosis, but unlikely neurocysticercosis [28].

Since only 119 of the *T. solium *sequences described in this work showed similarity to *T. solium *Genome Project sequences, our results significantly contribute to the knowledge of the parasite expression profile by increasing the number of sequenced transcripts and through functional annotation of several genes. Thus, the present report is complementary to the *T. solium *genome initiative and may be helpful on the parasite genome assembly and annotation [29], as well as on studies of intra- and inter-specific sequence variability.

Considering the overall picture of the *T. solium *cysticerci transcriptome presented in this work, comparative sequence analysis revealed 350 sequences (43%) producing hits with a database. Despite the small dataset, it is interesting to note that Aguilar-Diaz *et al*. [1] found a very similar picture in the analysis of the transcriptome of adult worms, with 40% of the genes showing no hits. A systematic, functional investigation of these unknown genes using postgenomic tools such as "gene knockout" or RNA-mediated "knockdown" is desirable.

Several protein domains related to cell structure, including cell wall organization, were found among the generated sequences. The pistil-specific extensin-like and the vinculin/alpha-catenin motifs found in this study are of special interest due their role in cell wall structure and interaction. According to Interpro, the pistil-specific extensin-like protein motif [Interpro:IPR003882] is frequently found in the cell-wall proteins of many plants, and can account for up to 20% of their dry weight. Interestingly, this motif is also found in metazoans like *Brugia malayi *[Interpro:A8Q5T0/A8QDB8] and *C. elegans *[Interpro:Q20327]. Since extensin-like proteins in plants are involved in cell wall strengthening in response to mechanical stress, such as attack by pests or plant-bending in the wind, it is reasonable to hypothesize a similar role on the *T. solium *cysts walls, conferring rigidity with a possible role in parasite defense.

The vinculin and/or alpha-catenin are eukaryotic actin-binding protein motifs, usually containing proline-rich motifs and several ligand-biding motifs. Vinculins are frequently used as markers for cell-cell and cell-extracellular matrix junctions, named as focal adhesions, also interacting with other structural proteins such as talin and alpha-actinins [30]. It is tempting to speculate that proteins containing these motifs may have a function on the organization of the cysticerci wall as well as on the interaction with host's tissues.

Oxidative and other types of stress are inherent to the host environment to which a parasite is exposed. Therefore, proteins that allow the cysticerci to cope with stress may be important in infection maintenance. In this study several heat shock proteins (hsp16, hsp20, hsp25, hsp70, hsp86, and hsp90) and other stress response-related proteins have been identified as being transcribed by this developmental stage. Previous studies with *T. solium *cysticerci showed that the expression of 70 and 80 kDa heat shock proteins was highly induced under temperature stress [31]. Recently, another heat shock protein of 35.4 kDa was described for *T. solium *cysticerci and points out the importance of such proteins for the parasite life cycle [32].

The host immune response to tissue parasitism is an important aspect to the establishment and development of the neurocysticercosis pathology. In this study, the 'heat shock 90 kDa' protein and the 'anaphylatoxin-like domain' (a complement-associated protein in vertebrates) – which are described for the first time for the *T. solium *cysticerci – have been associated to a possible immune response in invertebrates [33,34] and along with the host immunity may be involved on the host-parasite immunological interplay.

Among several genes related to the antigenic coat of the parasite, the TEG-Tsol gene is of major importance for both immune diagnostic and vaccine development, due to its high antigenicity, strong similarity (~97%) between *T. solium *and *T. saginata *paralogs, conservation among other taeniid species and reactivity to distinct animal sera [20]. TEG-Tsol was found among the ORESTES in the present study and corresponds to the major protoscolex surface antigen detected in *E. granulosus *(EG10) and *E. multilocularis *(EM10) [35], which is also expressed in the oncospheres and on adult tapeworm tegument of both *T. solium *and *T. saginata*, as well as on the tegument of the *T. solium *cysticerci [36-38].

Despite some encouraging results on vaccine development [39-41], several studies have pointed out intra- and inter-specific variability of taeniid species at both genotypic and phenotypic levels [26,42-50], which may represent a problem for the global-scale use of single- or multi-antigen recombinant vaccines. Thus, genome and transcriptome sequences – especially when derived from parasites collected at different endemic areas – are of major importance to address such variability and to point new vaccine and diagnostic/prognostic candidate markers. In this context, differently from genomic markers, ESTs are powerful tools not only to indicate potentially relevant candidates, but also to provide experimental evidence of expression specific developmental stages.

## Conclusion

The sequencing effort presented here is complementary to the *T. solium *Genome Project, having described several unknown genes for this species, which may have direct and immediate applications on diagnosis, therapeutics and/or vaccine development. Furthermore, this database represents part of a key resource to understanding aspects of the cysticerci biology and host/parasite interaction. Considering the ongoing efforts to sequence the hydatid disease agents (*E. granulosus *and *E. multilocularis*) along with the *T. solium *Genome Project [1,28], we hope our results can contribute to the development of comparative parasitic metazoan genomics, yielding new molecular diagnosis targets [51] and new insights into the pathogenesis of cysticercosis and taeniasis.

## Methods

### Cysticerci collection

*Taenia solium *cysticerci were collected from a naturally infected, landrace, bred pig (*Sus scrofa*). The animal was humanely sacrificed and cysticerci, spontaneously detached from abdominal and thoracic muscles were recovered and carefully micro-dissected to remove any tissue fragments that remained attached. Cysts were extensively washed with phosphate-buffered saline and immediately stored at -80°C. The study was previously approved by the Ethics Committee on Animal Research of the Faculty of Animal Science and Food Engineering (FZEA) of Universidade de São Paulo (USP), and was carried out following the institution's guidelines for animal husbandry.

### RNA extraction, RT-PCR and cDNA libraries preparation

Total RNA was obtained from cysticerci using the Trizol^® ^(Invitrogen, Carlsbad). Messenger RNA (mRNA) was purified using the μMACs mRNA isolation kit (Miltenyi Biotec, Bergisch Gladbach), following manufacturer's directions, as described [52]. mRNA concentration was evaluated by spectrophotometry (U-3010 Hitachi, Tokyo, Japan) and 25 ng mRNA aliquots were frozen for the posterior generation of ORESTES amplification profiles as described [12]. Briefly, cDNA was synthesized and amplified with some of the oligonucleotide primers previously used in the *S. mansoni *transcriptome project [12]. Twenty cDNA mini-libraries were constructed using ORESTES and a set of different oligonucleotide primers (see Additional file 12). The amplification profiles were evaluated in ethidium bromide-stained agarose gels, cloned in pGEM-T-Easy plasmids (Promega Corporation, Madison, USA) and used for *Escherichia coli *(strain DH10β) transformation. Recombinant clones were obtained by selective growth (X-Gal, IPTG and ampicillin), screened by PCR amplification of the insert using primers pGEM-F (5'-ACG CCA AGC TAT TTA GGT GAC ACT ATA-3') and EXCEL-R (5'-GTT GTA AAA CGA CGG CCA GTG AAT-3') and stored as glycerol stocks at -80°C. For sequencing, the bacterial clones were grown in LB medium for 20 hours at 37°C, followed by plasmid DNA extraction by alkaline lysis according to standard protocols [53].

### DNA sequencing and analysis

ORESTES sequencing was carried out by two laboratories located at UFSC and USP using the DYEnamic^® ^ET Dye Terminator kit (GE Healthcare, Fairfield) or ThermoSequenase II dye terminator cycle sequencing kit (Amersham-Pharmacia Biotech) in a MegaBace 1000^® ^DNA Analysis System (GE Healthcare) and on a ABI PRISM^® ^3100 Genetic Analyzer (Applied Biosystems, Foster City), respectively. Briefly, each sequencing reaction used 5 pmol of pGEM-F or EXCEL-R oligonucleotides, and PCR products [54] or plasmid DNA as templates. The labeling conditions were: 95°C/25 sec., 35 cycles of 95°C/15 sec., 50°C/20 sec. and 60°C/90 sec. The products were then precipitated (70% isopropanol), injected at 2 KV for 100 sec. and electrophoresed for 140 min. at 7 KV.

Sequence analysis was performed using the STINGRAY system (System for Integrated Genomic Resources and Analysis), an improved version of the formerly published GARSA system (Genomic Analysis Resources for Sequence Annotation) [55]. Briefly, the system workflow initially performs evaluation of the quality of the obtained chromatograms (cut-off Phred ≥ 15) following removal of vector sequences through Phred and Cross-match [56,57] and then clustering the sequences using CAP3 [58]. Following similarity searches performed by Blast (Basic Local Alignment Tool), Psi-Blast (Position-Specific Integrated Blast), RPSBlast (Reverse Position-specific Blast) [59], InterProScan [60] and HMMER (Hidden Markov Models for sequence profile analysis) [61] packages against local pre-formatted databases, blast analysis was also performed using all EST sequences from the *T. solium *Genome Project and the *Sus scrofa *genome available at GenBank. After removal of ribosomal RNA (rRNA) sequences, blast analysis against the *Sus scrofa *genome was used to separate parasite sequences from host sequences, creating two datasets that were evaluated separately. Functional annotation was performed using the Gene Ontology (GO) vocabulary as described by Jones *et al*. [62] and putative sub-cellular localization of each coding sequence was performed through the Wolf-PSORT program [63]. The G+C content of singlets and clusters was estimated by the GeeCee program (EMBOSS – European Molecular Biology Open Software Suite – package) and the tRNA sequences were predicted by tRNAscan-SE [64].

The results were then individually and manually checked during annotation, when sequences were validated as CDS when presenting i) high similarity values (e-value < = e^-15 ^and similarity>75%) with protein databases (uniprot_swissprot, uniprot_trembl, uniref90, refseq_protein) or with protein sequences from phylogenetically related organisms (Cestoda and/or Trematoda) available on GenBank, ii) the presence of conserved domains as revealed by RPS-Blast against CDD (see Additional file 6), COG (see Additional file 7) and KOG databases (see Additional file 8); iii) the presence of protein domains as revealed by InterProScan and HMMER and iv) annotations on Gene Ontology analysis, when available. *T. solium *sequences having no protein domain and showing exclusive hits with high similarity values (e-value < = e^-15 ^and similarity>75%) with 'hypothetical proteins' or 'hypothetical conserved proteins' from GenBank were annotated accordingly.

The *T. solium *cysticerci annotated transcripts, the host-parasite transcribed sequences, all databases used for comparative analysis as well as the additional material to this work are available online at the STINGRAY system .

## Abbreviations

ESTs: Expressed Sequence Tags; ORESTES: Open Reading frame Expressed Sequence Tags; PCR: Polymerase Chain Reaction.

## Competing interests

The authors declare that they have no competing interests.

## Authors' contributions

CRA and PHS are the main authors. CRA, PHS, GW, AAMM, ECG and EDN have equally contributed to this work. TCMS, GR, EBS, JBR, MMS, AZ, HBF, KT and AMRD have participated on the sequence analysis. All authors have participated on the manuscript preparation.

## Authors' informations

GW, EBS, GR and MMS are recipients of CNPq scholarships. PHS and TCMS are recipients of CAPES scholarships. ECG is currently a CNPq Post-Doctoral Fellow at BMRC/UEA, UK. EDN is a visiting scientist at MD Anderson Cancer Center, Houston TX, USA.

## Supplementary Material

Additional file 1 **Table S1**. List of *Taenia solium *ESTs and their GenBank accession numbers. Click here for file

Additional file 2 **Table S3**. Distribution of the *Taenia solium *EST according to their characteristics and similarity analysis. Click here for file

Additional file 3 **Table S4**. Number of *Taenia solium *ORESTES according to their annotation as coding sequences (CDS). Click here for file

Additional file 4 **Table S5**. Distribution of *Taenia solium *EST hits according to the Gene Onthology (GO) classification. Click here for file

Additional file 5 **Table S6**. *Taenia solium *ESTs with predicted extra-cellular sub-localization by Wolf-PSORT. Click here for file

Additional file 6 **Table S7**. Number of *Taenia solium *EST eliciting hits with the CDD database. Click here for file

Additional file 7 **Table S8**. Number of *Taenia solium *EST eliciting hits with the COG database. Click here for file

Additional file 8 **Table S9**. Number of *Taenia solium *EST eliciting hits with the KOG database. Click here for file

Additional file 9 **Picture S1**. Distribution of the most frequent Eukaryotic Orthologous Groups categories observed for the *Taenia solium *sequences. Click here for file

Additional file 10 **Table S10**. List of *Taenia solium *EST with similarity to the parasite genome project sequences. Click here for file

Additional file 11 **Table S11**. List of interesting genes found in *Taenia solium *ESTs. Click here for file

Additional file 12 **Table S2**. List of cDNA mini-libraries, primers used for generation of the *Taenia solium *EST profiles and the number of sequences obtained in each library. Click here for file
