# Supplementary material for: Transcriptome analysis of Taenia solium cysticerci using Open Reading Frame ESTs (ORESTES)
Source: Parasit Vectors. 2009 Jul 31;2:35. doi: 10.1186/1756-3305-2-35 (PMC2731055; doi:10.1186/1756-3305-2-35)
Supplement: Additional file 9 — Picture S1. Distribution of the most frequent Eukaryotic Orthologous Groups categories observed for the Taenia solium sequences. [file 1756-3305-2-35-S9.ppt]

## Slide 1
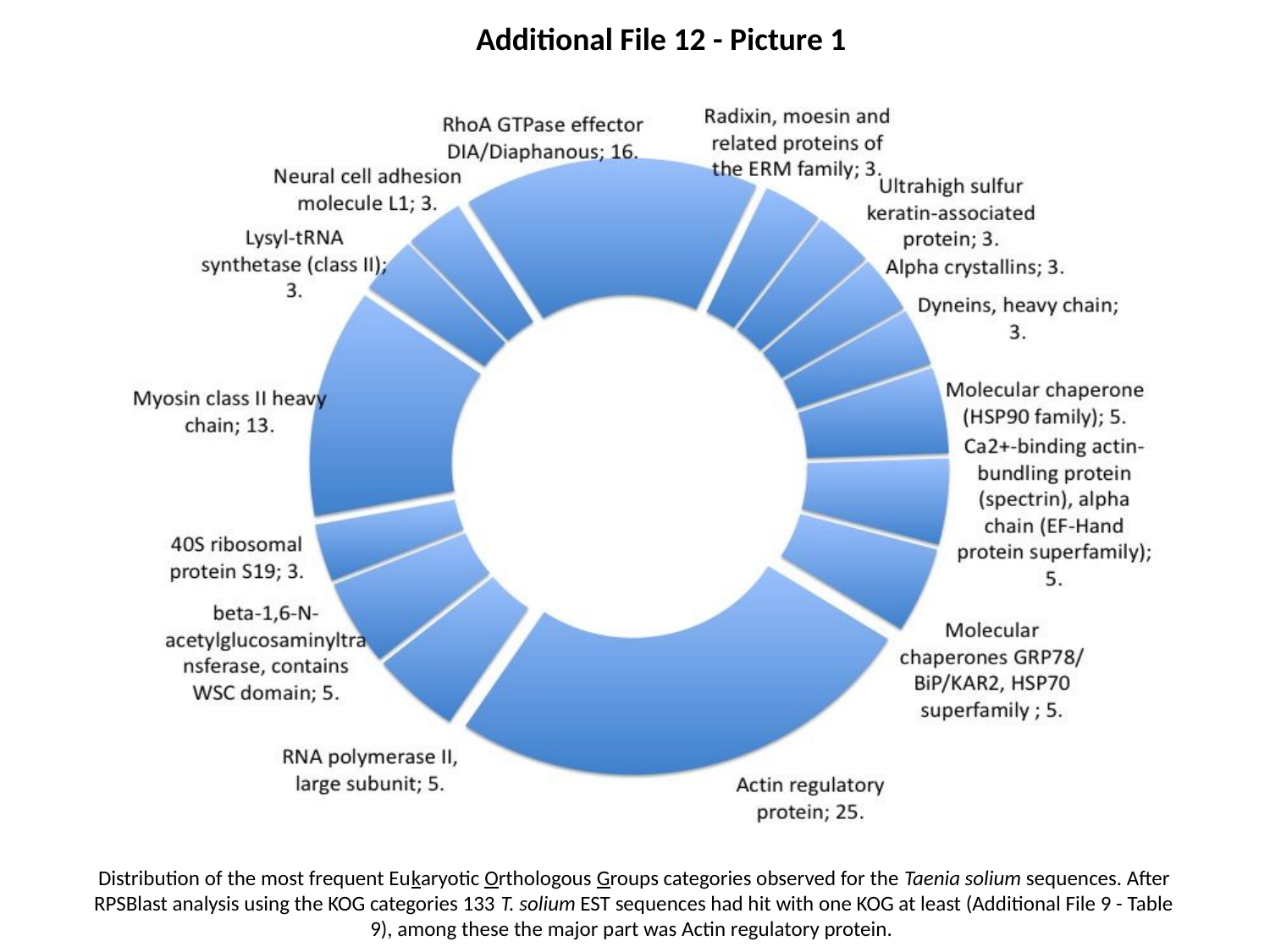

Additional File 12 - Picture 1
Distribution of the most frequent Eukaryotic Orthologous Groups categories observed for the Taenia solium sequences. After RPSBlast analysis using the KOG categories 133 T. solium EST sequences had hit with one KOG at least (Additional File 9 - Table 9), among these the major part was Actin regulatory protein.
